# 

**Published:** 1887-05

**Authors:** 


### OUR ADVERTISERS.

During the recent meeting of the State Medical Association in this city, there were a number of houses which made handsome exhibits of their goods.

R. A. Robinson & Co., of Louisville, had a very creditable display of pharmaceutical preparations. Among them were their syrup of hypophosphites and their elixir of paraldehyd, a new hypnotic and anodyne, samples of each were freely distributed among the physicians.

Fairchild, Brother & Foster's exhibit was very fine, and their representative was kept busy demonstrating the value of their pepsin as compared with that of other manufacturers. It is safe to say he convinced every one of the value of Fairchild's digestive ferments.

Budwell & Christian had an exhibition of their emulsion of pure cod liver oil with the iodides. They claim for this preparation virtues that no other emulsion has. They will be pleased to send samples of it to any physician writing for same.

The Galvano Faradic Manufacturing Co., of New York, had a fine display of batteries at the hall. We are informed that these goods will hereafter be on sale by C. O. Tyner, of this city, at No. 30 Marietta street.

Doliber, Goodale & Co., of Boston, proprietors of Mellin's food for infants and invalids, had a large amount of this food on exhibition, and it required the constant attention of two men to answer the many questions asked about it and to give out samples.

Parke, Davis & Co., of Detroit, had the largest display of any of the exhibitors, consisting of a general line of their fluid extracts and normal liquids. These gentlemen have succeeded in building up for their various preparations an enviable reputation.

NESTLE'S MILK FOOD.—We desire to direct attention to the advertisement of this food to be found elsewhere in THE JOURNAL. It is well endorsed by those who have used it. Send for samples of it and give it a trial.

